# Transcriptomic response of primary human airway epithelial cells to flavoring chemicals in electronic cigarettes

**DOI:** 10.1038/s41598-018-37913-9

**Published:** 2019-02-01

**Authors:** Hae-Ryung Park, Michael O’Sullivan, Jose Vallarino, Maya Shumyatcher, Blanca E. Himes, Jin-Ah Park, David C. Christiani, Joseph Allen, Quan Lu

**Affiliations:** 1 Department of Environmental Health, Harvard T.H. Chan School of Public Health, Boston, 02115 Massachusetts USA; 2 Department of Biostatistics, Epidemiology and Informatics, University of Pennsylvania, Philadelphia, Pennsylvania 19104 USA; 3 Department of Genetics and Complex Diseases, Harvard T.H. Chan School of Public Health, Boston, 02115 Massachusetts USA

## Abstract

The widespread use of electronic cigarettes (e-cigarettes or e-cig) is a growing public health concern. Diacetyl and its chemical cousin 2,3-pentanedione are commonly used to add flavors to e-cig; however, little is known about how the flavoring chemicals may impair lung function. Here we report that the flavoring chemicals induce transcriptomic changes and perturb cilia function in the airway epithelium. Using RNA-Seq, we identified a total of 163 and 568 differentially expressed genes in primary normal human bronchial epithelial (NHBE) cells that were exposed to diacetyl and 2,3-pentanedione, respectively. DAVID pathway analysis revealed an enrichment of cellular pathways involved in cytoskeletal and cilia processes among the set of common genes (142 genes) perturbed by both diacetyl and 2,3-pentanedione. Consistent with this, qRT-PCR confirmed that the expression of multiple genes involved in cilia biogenesis was significantly downregulated by diacetyl and 2,3-pentanedione in NHBE cells. Furthermore, immunofluorescence staining showed that the number of ciliated cells was significantly decreased by the flavoring chemicals. Our study indicates that the two widely used e-cig flavoring chemicals impair the cilia function in airway epithelium and likely contribute to the adverse effects of e-cig in the lung.

## Introduction

Electronic cigarettes (e-cigarettes or e-cig) have gained popularity as a substitute for cigarettes or cigars, and they have been a gateway to cigarettes for those who never smoked^1^. The World Health Organization (WHO) reported that the global market for Electronic Nicotine and Non-Nicotine Delivery Systems in 2015 was estimated to be $10 billion and about 56% was accounted by the United States of America^2^. As the popularity and use of e-cig continue to increase, there is an urgent need to assess comprehensively the chemical compositions and potential health effects of e-cig. Although concerns regarding e-cig primarily focus on exposure to nicotine^3–6^, e-cig contains myriad chemicals, including carbonyl compounds, aldehydes, fine particulate matter, metals, propylene glycol, glycerol, formaldehyde, and volatile organic compounds (VOCs)^7–16^. More recently, Allen *et al*.^17^ showed that flavoring chemicals are in over 90% of commercially marketed flavored e-cig.

The most common flavoring compound added to e-cig is diacetyl^15,16^, which is a small, volatile compound with butter-like aroma^18^. In the early 2000s, a series of studies reported a strong association between the production of butter-flavored microwave popcorn and bronchiolitis obliterans (“popcorn lung”), an irreversible lung disease^17,19^. Diacetyl was the most prominent chemical in the butter flavorings and its effect was dose-dependent^20–23^. Likely because of the known link between diacetyl and “popcorn lung”, 2,3-pentanedione, which has similar flavor properties to diacetyl, was used as a substitute in some e-cig^24^. Indeed, Allen *et al*. found 2,3-pentanedione in nearly 50% of e-cig tested in 2016^17^. Despite the widespread use of these flavoring chemicals in e-cig, there is a paucity of studies on potential adverse effects of these chemicals to e-cig users.

Some earlier rodent studies have shown that exposures to diacetyl or 2,3-pentanedione damage airway epithelium in mice and rats^25–29^. However, the mechanism underlying the adverse effects is not known; it is also not known whether the flavoring chemicals similarly damage human airway epithelium. In this study, we utilized primary normal human bronchial epithelial (NHBE) cells that are cultured at an air-liquid interface (ALI) to mimic the *in vivo* airway characteristics^30^. Using RNA-Seq, we performed global transcriptomic profiling in primary NHBE cells exposed to diacetyl or 2,3-pentanedione. Our data showed that diacetyl and 2,3-pentanedione induced significant transcriptomic changes, including those related to ciliogenesis, in the primary NHBE cells. Consistent with the transcriptomic data, diacetyl or 2,3-pentanedione-treatment decreased the number of ciliated cells in differentiated NHBE cell population, indicating an impairment of ciliogenesis in airway epithelium by the flavoring chemicals in e-cig.

## Results

### Transcriptomic profiling of human bronchial epithelial cells exposed to flavoring chemicals

**Figure 1:**
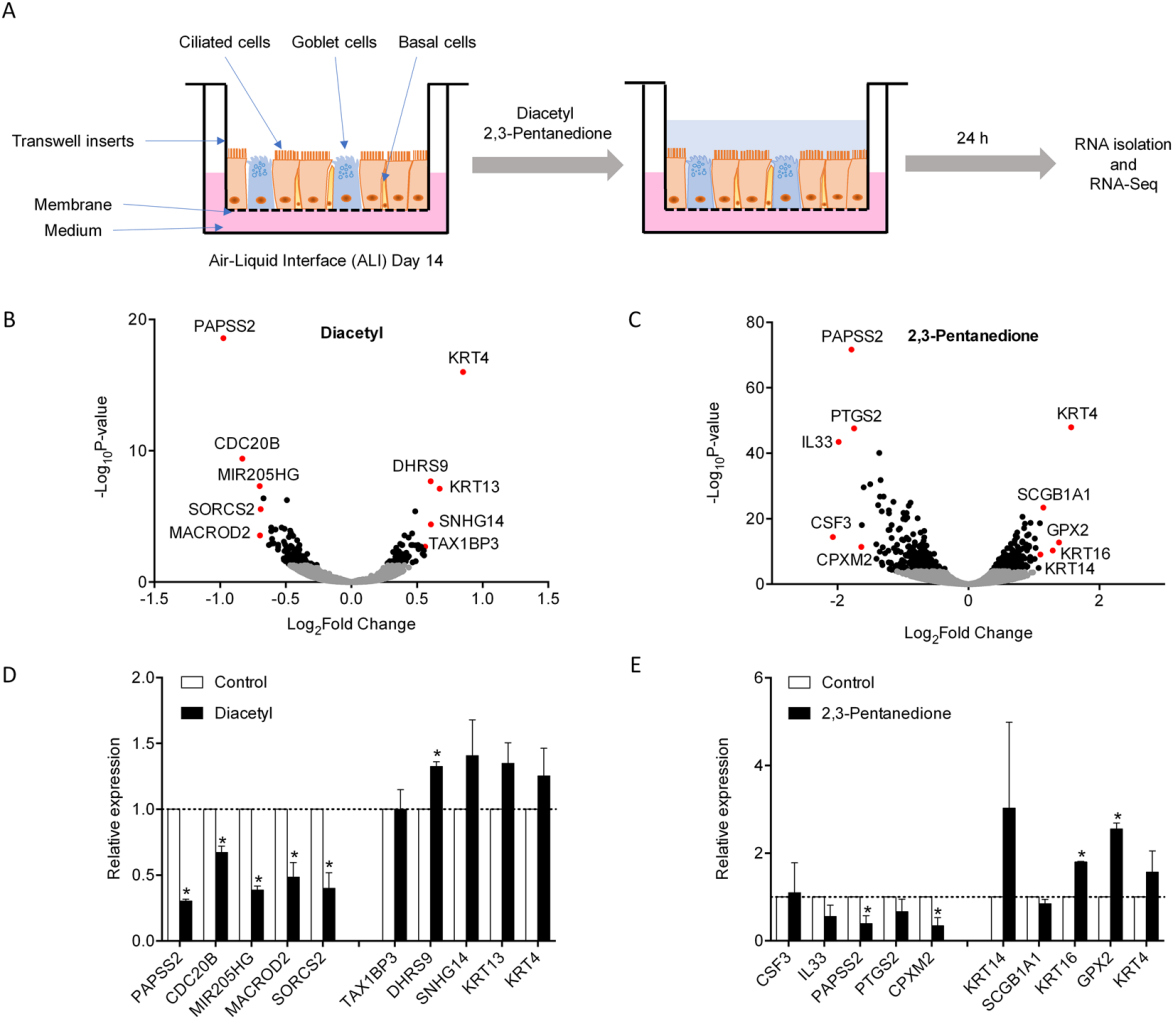
Identification of differential gene expression in NHBE cells exposed to flavoring chemicals by RNA-seq. (**A**) Schematic workflow of the study. (**B**,**C**) Volcano plot of RNA-seq results with top 10 genes annotated in diacetyl or 2,3-pentanedione-exposed NHBE cells for 24 h. Black dots represent gene with Padj < 0.05. Grey dots represent genes that do not meet the significance threshold. (**D**,**E**) qPCR validation of top 10 genes identified by RNA-seq with diacetyl or 2,3-pentanedione. *P < 0.05 compared to control. N = 3 subjects.

To examine the effect of flavoring compounds on human airway epithelium, we utilized air–liquid interface (ALI) cultures of primary NHBE cells. NHBE cells cultured under ALI after 14 days differentiate into a mixture of ciliated cells, goblet cells as well as some remaining basal cells (Fig. 1A), thus closely mimicking human airway epithelium *in vivo*^30^. We first exposed differentiated mature primary NHBE cells (ALI culture day 14) to diacetyl, 2,3-pentanedione, or vehicle control (H_2_O) for 24 h. The rationale for choosing 25 ppm diacetyl is that the previous study indicates that no observable adverse effect level for sub-chronic inhalation may be less than 25 ppm diacetyl^28^. In addition, extrapolation of the mouse dose-response relationship to humans suggested no sensory irritation to warn employees during acute diacetyl exposures at concentrations less than 20 ppm^31^. The concentration of 2,3-pentanedione was selected because the lowest concentration with observable phenotypes such as bronchial fibrosis, cell morphology, or lung resistance ranges from 100 ppm to 150 ppm *in vitro* and *in vivo*^29,32,33^. LDH (lactate dehydrogenase) assay confirmed that diacetyl and 2,3-pentanedione used at these concentrations did not induce significant cytotoxicity at 6 or 24 h (Supplementary Fig. S1). After 24-hour exposure to control, diacetyl, or 2,3-pentanedione, total RNAs from the cells were extracted and used for RNA-seq-based transcriptional profiling (Fig. 1A). We constructed RNA-seq libraries, each with a unique barcode that allows multiplexing. To minimize the variation of sequencing runs, we pooled barcoded RNA-seq libraries for next generation deep sequencing. We obtained an average of ~18 million reads per sample and tested for differential expression of GRCh37 Ensembl-annotated genes. Following stringent multiple testing corrections, we identified a total of 163 and 568 differentially regulated genes after exposure to diacetyl and 2,3-pentanedione, respectively, as shown in the volcano plots in Fig. 1B and C.

### qPCR validation of differentially regulated genes by flavoring chemicals

We ranked the differentially regulated genes for each treatment group by fold change. We then selected a total of 10 genes (five most upregulated and five most downregulated genes) with each treatment for qRT-PCR validation (Supplementary Tables S1 and S2). We treated ALI cultures of primary NHBE cells derived from three different donors with vehicle control, 25 ppm diacetyl, or 100 ppm 2,3-pentanedione. For diacetyl treatment, out of the top 10 differentially expressed genes, qRT-PCR verified 6 genes that were significantly changed compared to control (Fig. 1D). Diacetyl treatment suppressed the expression of *PAPSS2*, *CDC20B*, *MIR205HG*, *MACROD2*, and *SORCS2* and increased the expression of *DHRS9* (Fig. 1D. p < 0.05). Expression of *SNHG14*, *KRT13*, and *KRT4* was also increased with diacetyl treatment, but the changes were not statistically significant (Fig. 1D). For 2,3-pentanedione treatment, qRT-PCR confirmed changes in mRNA expression of *PAPSS2*, *CPXM2*, *KRT16*, and *GPX2* (Fig. 1E, p < 0.05).

### Functional Annotation Enrichment using DAVID

We then used the DAVID pathway analysis tool to identify ontological categories that were enriched among the differentially expressed genes induced by exposures to flavoring chemicals. For the diacetyl treatment gene dataset, pathways related to “Cell projection”, “Cilium”, “Intermediate filament protein, conserved site”, and “Cilium biogenesis/degradation” were significantly enriched (Table 1). For the 2,3-pentanedione treatment dataset, pathways related to “cell-cell adherens junction”, “Ciliopathy”, “cilium morphogenesis”, “Cilium biogenesis/degradation”, “Dynein”, and “Cytoskeleton” were highly enriched (Table 2). The full list of enriched terms is available in Supplementary Tables S3 and S4. Interestingly, pathways related to cilium and cilium biogenesis/degradation, were highly enriched in both diacetyl and 2,3-pentanedione datasets.

**Table 1:** Enriched terms in the gene list differentially regulated by diacetyl by DAVID analysis.

Annotation Cluster 1	Enrichment Score: 4.6		
**Category**	**Term**	**Genes**	Padj*
UP_KEYWORDS	Cell projection	20	4.6E-04
UP_KEYWORDS	Cilium	11	4.3E-04
**Annotation Cluster 2**	**Enrichment Score: 3.4**		
**Category**	**Term**	**Genes**	**Padj**
INTERPRO	IPR018039:Intermediate filament protein, conserved site	7	0.003
UP_SEQ_FEATURE	region of interest:Coil 2	7	0.011
UP_SEQ_FEATURE	region of interest:Linker 12	7	0.011
UP_SEQ_FEATURE	region of interest:Linker 1	7	0.009
UP_SEQ_FEATURE	region of interest:Coil 1A	7	0.009
UP_SEQ_FEATURE	region of interest:Coil 1B	7	0.009
UP_SEQ_FEATURE	region of interest:Rod	7	0.006
UP_KEYWORDS	Intermediate filament	7	0.002
UP_SEQ_FEATURE	region of interest:Head	7	0.005
INTERPRO	IPR001664:Intermediate filament protein	7	0.005
SMART	SM01391:SM01391	7	0.004
INTERPRO	IPR009053:Prefoldin	5	0.005
UP_SEQ_FEATURE	site:Stutter	5	0.025
GOTERM_CC_DIRECT	GO:0005882~intermediate filament	7	0.012
UP_SEQ_FEATURE	region of interest:Tail	6	0.045
**Annotation Cluster 3**	**Enrichment Score: 2.32**		
**Category**	**Term**	**Genes**	**Padj**
UP_KEYWORDS	Cilium biogenesis/degradation	8	0.007

*Padj: adjusted p-values for multiple comparisons by the Benjamini Hochberg correction.

**Table 2:** Enriched terms in the gene list differentially regulated by 2,3-pentanedione by DAVID analysis.

Annotation Cluster 1	Enrichment Score: 12.39		
**Category**	**Term**	**Genes**	Padj*
GOTERM_CC_DIRECT	GO:0005913~cell-cell adherens junction	40	6.9E-12
GOTERM_MF_DIRECT	GO:0098641~cadherin binding involved in cell-cell adhesion	38	6.1E-11
GOTERM_BP_DIRECT	GO:0098609~cell-cell adhesion	33	6.7E-08
**Annotation Cluster 2**	**Enrichment Score: 5.13**		
**Category**	**Term**	**Genes**	**Padj**
UP_KEYWORDS	Ciliopathy	16	4.8E-05
GOTERM_BP_DIRECT	GO:0060271~cilium morphogenesis	17	0.003
GOTERM_BP_DIRECT	GO:0042384~cilium assembly	15	0.012
UP_KEYWORDS	Cilium biogenesis/degradation	15	0.001
UP_SEQ_FEATURE	region of interest:AAA 6	7	3.6E-04
COG_ONTOLOGY	Cytoskeleton	8	2.0E-04

*Padj: adjusted p-values for multiple comparisons by the Benjamini Hochberg correction.

**Figure 2:**
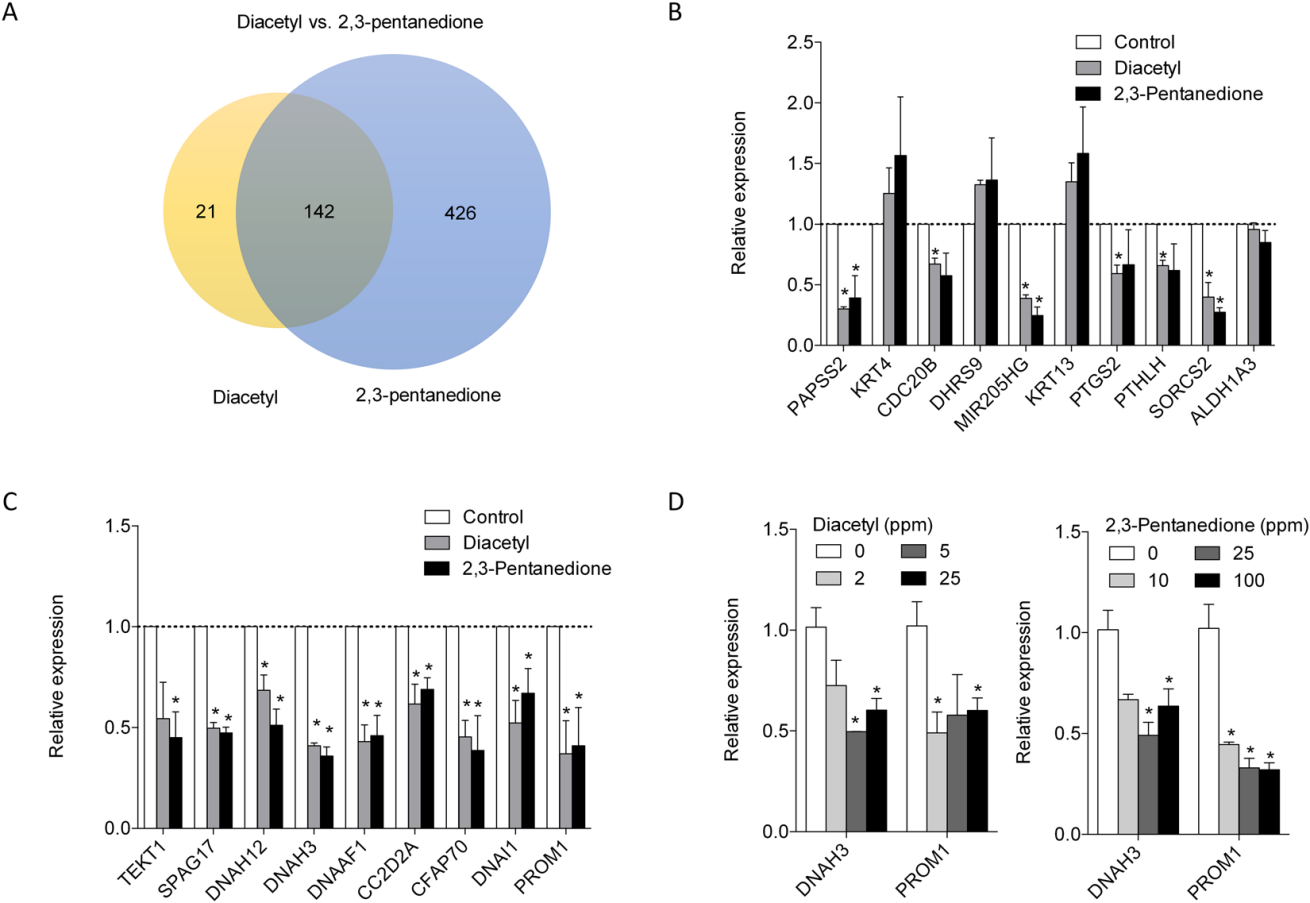
The transcriptomes of diacetyl and 2,3-pentanedione-exposed NHBE cells are highly overlapped. (**A**) The Venn diagram shows the overlap of differentially expressed gene sets (Padj < 0.05). (**B**) qPCR validation of the top 10 overlapped gene set with diacetyl and 2, 3-pentanedione treatment. *P < 0.05 compared to control. N = 3 subjects. (**C**) Expression of genes involved in cilia processes measured by qPCR. *P < 0.05 compared to control. N = 3 subjects. (**D**) Expression of cilia-involved genes in HNBE cells exposed to diacetyl or 2,3-pentanedione at varying concentrations. P < 0.05 compared to control.

The identification of common pathways by the individual DAVID pathway analyses of the transcriptomic datasets is consistent with the chemical similarity between diacetyl (chemical synonym: 2,3-butanedione) and 2,3-pentanedione. We further compared the lists of differentially regulated genes with the two flavoring chemicals. Among 163 differentially regulated genes with diacetyl, 142 genes (~87%) were also differentially expressed with 2,3-pentanedione treatment (Fig. 2A). We ranked these 142 genes according to the p-values and then used qRT-PCR to validate the top 10 genes (*PAPSS2, KRT4, CDC20B, DHRS9, MIR205HG, KRT13, PTGS2, PTHLH, SORCS2*, and *ALDH1A3*) in ALI-cultured NHBE cells from three different donors (Table 3). As shown in Fig. 2B, the expression profiles of these top 10 genes in response to diacetyl and 2,3-pentanedione treatments were very similar in terms of the direction and magnitude of fold changes. Among the ten genes, *PAPSS2, MIR205HG*, and *SORCS2* were significantly down-regulated by diacetyl or 2,3-pentanedione treatment (Fig. 2B, p < 0.05). *CDC20B, PTGS2*, and *PTHLH* were downregulated with diacetyl treatment (Fig. 2B, p < 0.05). DAVID analysis of the overlapping 142 genes in the two gene lists identified several significantly enriched pathways related to “Intermediate filament protein, conserved site”, “Extracellular space”, “Cilium”, “Cell projection”, and “Dynein” (Table 4), again reinforcing the notion that the two related flavoring chemicals may act on bronchial epithelium via common mechanisms. The full list of enriched terms is available in Supplementary Table S5.

**Table 3:** Top 10 genes differentially regulated in both diacetyl and 2,3-pentanedione treatment*.

Gene	Diacetyl		2,3-Pentanedione	
	Fold Change	Padj	Fold Change	Padj
*PAPSS2*	0.51	2.68E-25	0.29	1.16E-81
*KRT4*	1.80	2.70E-19	2.96	2.10E-72
*CDC20B*	0.56	1.01E-16	0.39	8.08E-41
*DHRS9*	1.52	4.04E-10	1.60	3.65E-11
*MIR205HG*	0.62	2.13E-08	0.33	2.80E-31
*KRT13*	1.59	4.87E-08	2.13	1.81E-19
*PTGS2*	0.30	1.15E-48	0.30	1.15E-48
*PTHLH*	0.71	4.14E-07	0.62	4.03E-14
*SORCS2*	0.62	5.79E-07	0.54	1.08E-09
*ALDH1A3*	1.40	2.85E-06	1.46	3.85E-10

*The gene list with diacetyl treatment was ranked by Padj values, then top 10 genes shared with 2, 3-pentanedione treatment were selected.

**Table 4:** Enriched terms in the gene list differentially regulated by both diacetyl and 2,3-pentanedione.

Annotation Cluster 1	Enrichment Score: 3.5		
**Category**	**Term**	**Genes**	Padj*
INTERPRO	IPR018039:Intermediate filament protein, conserved site	7	0.001
UP_SEQ_FEATURE	region of interest:Coil 2	7	0.004
UP_SEQ_FEATURE	region of interest:Linker 12	7	0.004
UP_SEQ_FEATURE	region of interest:Coil 1B	7	0.004
UP_SEQ_FEATURE	region of interest:Coil 1A	7	0.004
UP_SEQ_FEATURE	region of interest:Linker 1	7	0.004
UP_SEQ_FEATURE	region of interest:Rod	7	0.003
UP_KEYWORDS	Intermediate filament	7	0.001
UP_SEQ_FEATURE	region of interest:Head	7	0.002
INTERPRO	IPR001664:Intermediate filament protein	7	0.002
SMART	SM01391:SM01391	7	0.001
INTERPRO	IPR009053:Prefoldin	5	0.003
UP_SEQ_FEATURE	site:Stutter	5	0.014
GOTERM_CC_DIRECT	GO:0005882~ intermediate filament	7	0.007
UP_SEQ_FEATURE	region of interest:Tail	6	0.022
UP_KEYWORDS	Keratin	7	0.022
**Annotation Cluster 2**	**Enrichment Score: 1.98**		
**Category**	**Term**	**Genes**	**Padj**
GOTERM_CC_DIRECT	GO:0005615~extracellular space	22	0.010
**Annotation Cluster 3**	**Enrichment Score: 1.89**		
**Category**	**Term**	**Genes**	**Padj**
UP_KEYWORDS	Cilium	11	0.000
UP_KEYWORDS	Cell projection	18	0.001
UP_KEYWORDS	Dynein	4	0.046

*Padj: adjusted p-values for multiple comparisons by the Benjamini Hochberg correction.

### Expression of genes involved in cilia processes

Although our DAVID analysis identified “Intermediate filament” as one of the most significantly enriched terms by exposure to both flavoring chemicals in NHBE cells (Table 4), expression of genes included in the term (*KRT4*, and *KRT13*, and *KRT14*) was not validated with our initial qPCR validation of top hits (Fig. 1D,E). Because the “Cilium” pathway has the lowest p-value in the combined analysis (Table 4) and has been identified as a shared pathway in the individual analysis of the two datasets (Tables 1 and 2), we focused our follow up characterization on the genes involved cilium biogenesis. Our RNA-seq data showed that expression of at least 11 genes related to cilium (*TEKT1, CFAP70, PROM1, DNAH12, DNAI1, DNAH3, DNAAF1, CC2D2A, CFAP221, SPAG17*, and *DNAH6*) was significantly down-regulated in NHBE cells treated with 25 ppm diacetyl or 100 ppm 2,3-pentanedione (Supplementary Table S6). Using qRT-PCR, we validated the down-regulation of these genes in NHBE cells from three different donors upon either diacetyl or 2,3-pentanedione treatment (Fig. 2C). Consistent with RNA seq data, mRNA expression of *TEKT1, CFAP70, PROM1, DNAH12, DNAI1, DNAH3, DNAAF1, CC2D2A*, and *SPAG17* was significantly down-regulated with diacetyl and 2,3-pentanedione (Fig. 2C, p < 0.05) (*CFAP221* and *DMAH6* were excluded because specific primers were not available), suggesting that the flavoring chemicals may impair cilia biogenesis and functions. We measured expression of *DNAH3* and *PROM1*, two most down-regulated genes with flavoring chemicals (Fig. 1C), in NHBE cells exposed to flavoring chemicals at lower levels. Their expression was down-regulated by treatment with diacetyl and 2,3-pentanedione at levels as low as 2 and 10 ppm, respectively (Fig. 2D).

### Effect of diacetyl and 2,3-pentanedione on cilia biogenesis

**Figure 3:**
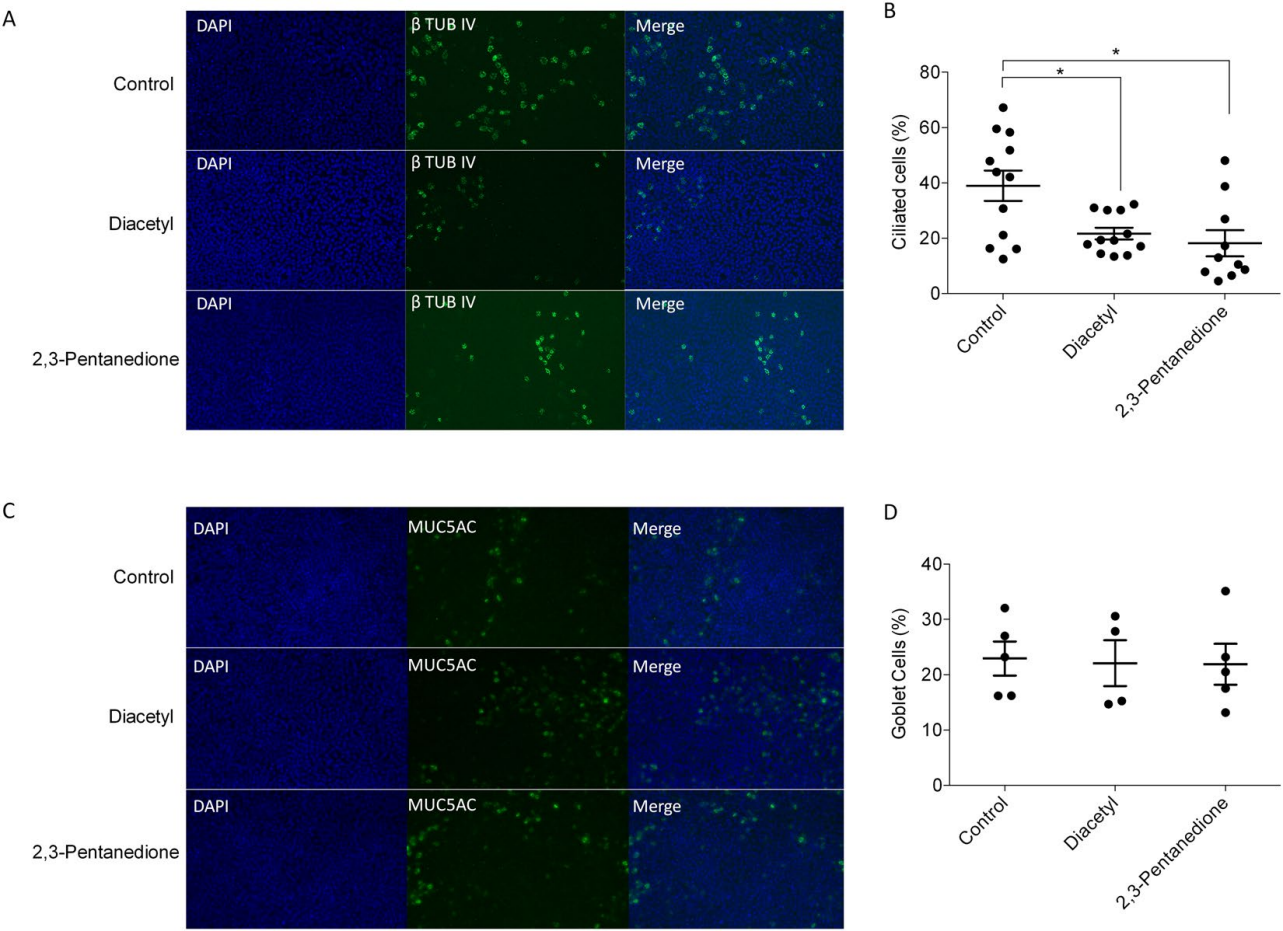
Immunofluorescence staining. NHBE cells at ALI day 14 were exposed to diacetyl (25 ppm) or 2,3-pentanedione (100 ppm) for 48 h and stained for β-tubulin IV (a marker for ciliated cells) or MUC5AC (a marker for goblet cells). (**A**) The first panel shows nuclei visualized by DAPI staining. The second panel shows ciliated cells stained for β-tubulin IV. Representative images are shown. (**B**) The number of ciliated cells was normalized to the number of total cells in the field of view. Each dot represents a single image. *P < 0.05 compared to control. N = 1–3 subjects. (**C**) The first panel shows nuclei visualized by DAPI staining. The second panel shows goblet cells stained for MUC5AC. Representative images are shown. (**D**) The number of goblet cells was normalized to the number of total cells in the field of view. Each dot represents a single image. *P < 0.05 compared to control. N = 3 subjects.

To further investigate the effect of flavoring chemicals on cilia biogenesis, we exposed ALI-cultured NHBE cells to diacetyl or 2,3-pentanedione for 48 h and performed β-tubulin IV immunofluorescence staining for ciliated cells. The number of ciliated cells was normalized to the number of total cells in the field of view. Fig. 3A shows DAPI nuclei staining, β-tubulin IV staining, and the merged images, respectively. As shown in Fig. 3A and B, the number of ciliated cells was significantly decreased by diacetyl and 2,3-pentanedione treatment, suggesting that both flavoring chemicals may target ciliated cells disrupting cilia biogenesis. To test whether flavoring chemicals affect other cell types in the epithelium, we stained for MUC5AC, which is a marker for goblet cells, another major cell type in lung epithelium. Either diacetyl or 2,3-pentanedione did not affect the number of goblet cells after 48-h exposure (Fig. 3C and D), suggesting that ciliated cells may be more susceptible to the effect of flavoring chemicals than goblet cells.

## Discussion

The use of e-cig has outpaced the scientific studies examining the potential effects of e-cig and its chemical components. Although chemicals such as diacetyl and its substitute 2,3-pentanedione are frequently used and found in commercial e-cig products to add flavor, little is known about how these flavoring chemicals may impair lung function, in particular that of the airway epithelium, which is the first line of defense in the lung and is the direct target of the chemicals. Here we show that both diacetyl and 2,3-pentanedione induce significant transcriptomic changes, including those related to ciliogenesis, and decrease the number of ciliated cells, potentially impairing the cilia function of primary human airway epithelial cells. These data suggest that 2,3-pentanedione may not be a safe replacement of diacetyl, but may have similar characteristics to that of diacetyl, a flavoring compound associated with the development of “popcorn lung”^23^. Therefore, this study calls for strict assessment of potential toxicity of 2,3-pentanedione as well as diacetyl.

We identified that both diacetyl and 2,3-pentanedione led to significant transcriptomic changes related to ciliogenesis in primary human airway epithelial cells, which may impair the cilia function. Ciliated cells are predominant within human airways constituting 50%–80% of airway epithelial lining and play an important role in the mucociliary transport^34–36^. Cilia function can be modulated by ciliary beating, the length of the cilia, the ratio of ciliated to non-ciliated areas, the structure and orientation of cilia, mucus lining of the epithelium, and exposure to endogenous and exogenous factors^37–48^. Impaired cilia function has been associated with lung diseases such as COPD and asthma^34,37,49–52^. Our study has shown that exposure to flavoring chemicals leads to decreased number of ciliated cells with concomitant down-regulation of cilia-related genes in ALI culture system of NHBE cells *in vitro*, mimicking the *in vivo* airway characteristics with tight junctions and a differentiation state with ciliated, basal, and secretory cells^30,53^. This is consistent with rodent studies showing that diacetyl or 2,3-pentanedione treatment resulted in airway epithelial injury characterized by flattening of cells, loss of microvilli and cilia, and fissure formation^25,27,54^. In addition, the proteomics study by Foster *et al*. (2017) reported down regulation of cilia-related proteins including DNAI1, that was also significantly down-regulated in our study, and a loss of ciliated cells in diacetyl-exposed human airway epithelial cells. Further research is needed to determine the structural-functional regulation of cilium by exposure to flavoring chemicals and how this might relate to the initiation or progression of lung diseases.

In addition to transcriptomic changes related to ciliogenesis, our ontological enrichment analysis reveals that diacetyl and 2,3-pentanedione activate gene sets involved in cytoskeletal structure and function including keratins. Keratins are the typical intermediate filament proteins of epithelia and are important for the mechanical stability and integrity of epithelial cells and tissues. Moreover, some keratins also have regulatory functions and are involved in intracellular signaling pathways including apico-basal polarization, motility, cell size, protein synthesis and membrane traffic and signaling^55,56^. According to our RNA-Seq results, expression of *KRT4, KRT13, KRT14, and KRT16* were upregulated with diacetyl or 2,3-pentanedione treatment (Tables 1 and 2). Although the role of these keratins in lung diseases is not clear, further studies are necessary to evaluate their involvement on toxicity of flavoring chemicals in lung epithelium. Additionally, the interplay between cytoskeleton and ciliary function should be taken into consideration in future investigations because microtubule cytoskeletons of the cilium play critical roles in the regulation of cilium structure and function^44,57–62^.

Among the top 10 overlapping genes in the two flavoring chemicals, qPCR validated down-regulation of *PAPSS2* (3′phosphoadenosinse 5-phosphosulfate synthase 2), *MIR205HG* (MIR205 Host Gene), and *SORCS2* (Sortilin Related VPS10 Domain Containing Receptor 2), which do not belong to cilia or cytoskeleton pathways. As the first line of defense in the lung, bronchial epithelium metabolizes foreign compounds to facilitate the elimination of the foreign substance by a set of broad specificity enzymes capable of introducing new functional groups (Phase I reactions), or conjugating with charged molecules, to increase its water solubility (Phase II reactions)^63^. PAPSS mediates the synthesis of PAPS that is the universal sulfate donor for Phase II sulfation reaction^64^. Downregulation of *PAPSS* may lead to deficit in available sulfate donors and interfere with sulfation reactions. Therefore, exposure to the flavoring compounds may impair detoxifications of xenobiotics in the lung epithelium increasing susceptibility to toxicant exposure in the lung. In addition, previous studies reported the potential involvement of *PAPSS2* in lung carcinoma, showing that *PAPSS2* was down-regulated in in squamous cell lung carcinoma compared to normal lung tissues^65^. Similarly, miR-205 expression was down-regulated in lung cancer cell lines whereas its overexpression promoted an epithelial phenotype and inhibited tumor cell migration and metastasis formation in lung cancer models^66–68^. Furthermore, genetic polymorphisms of *SORCS2* have been associated with decreased survival in non-small cell lung cancer patients^69^. Although the present study implicates that exposure to flavoring chemical may impact airway epithelium through multiple mechanisms including the impaired cilia and cytoskeleton function and compromised detoxifications, further study is warranted on how these mechanisms alone or in combination may contribute to lung diseases such as bronchiolitis obliterans, lung cancer, COPD, and asthma.

Although this is the first whole transcriptomic profiling in NHBE cells exposed to flavoring chemicals, there are a few limitations. First, mechanisms through which flavoring chemicals impact ciliogenesis in lung airway epithelium are still unknown. It has been reported that α-dicarbonyl compounds including diacetyl and 2,3-pentanedione are highly reactive to cause protein cross-links^70^. We suggest that protein cross-linking or modifications by these flavoring chemicals may lead to inactivation of protein activities and disruption of protein-protein interactions, ultimately resulting in the dysregulation of signal-transduction pathways such as ciliogenesis. Studies on the transcription factors including Forkhead box J1 (FOXJ1), which regulate cilia pathways^71^, will further elucidate the mechanisms of adverse impacts by flavoring chemicals in lung epithelium. Second, the concentrations of flavoring chemicals in this study are much higher than the occupational limits for diacetyl and 2,3-pentanedione that are ranging from 5–10 ppb^17^. However, our study may be more relevant to acute exposure than chronic exposure because the popcorn workers had been exposed to diacetyl 2–40 ppm (up to 100 ppm)^72^ and it is suggested that the peak diacetyl exposure is a greater hazard than the time-weighted-average diacetyl exposure^27^. Third, NHBE cells were exposed to aqueous solution of flavoring chemicals that may not reflect real human exposure to e-cig. Further study using vapor exposure system will be warranted to confirm the results from this study^32,73^. Additionally, measuring concentrations of these chemicals in the medium overtime would be helpful to assess the actual concentration of these volatile chemicals in the medium. Last, we focused our investigation on diacetyl and 2,3-pentanedione because of our prior research from 2016 showing that these flavor chemicals are commonly found in e-cig^17^. However, it is possible that e-cig manufacturers have changed formulations since that time. This information can be difficult to confirm without constant lab testing because information on the exact flavoring formulation is not readily available in many cases. For example, one large e-cig producer lists on their website all of the ingredients in the products, but only uses the generic term ‘Flavors’ to describe the flavoring chemicals. Another lists ‘natural and artificial flavors’ on its package without listing specific ingredients. At least one large e-cig producer states on their website that they do not use diacetyl or 2,3-pentanedione, but they do not disclose what chemicals they are using to flavor their products. This is a critical information gap considering that, in addition to diacetyl and 2,3-pentanedione, there are additional 25 “High Priority” flavoring chemicals, according to a report on respiratory health concerns of flavoring chemicals produced by a flavoring chemical trade group^74^.

## Conclusions

Our findings reveal that two flavoring chemicals commonly found in e-cig, diacetyl and 2,3-pentanedione, induce similar transcriptional changes and affect biological pathways related to cellular morphology/integrity and cilium in NHBE cells. We further showed that exposure to diacetyl or 2,3-pentanedione down-regulated expression of cilia-related genes and decreased the number of ciliated cells. Because of the associations of diacetyl inhalation exposure and severe respiratory diseases and increasing popularity of e-cig use among people, further mechanistic studies are warranted to evaluate the effects of diacetyl and related flavoring compounds in e-cig on airway epithelium.

## Methods

### Cell culture and exposure

Normal Human Bronchial Epithelial (NHBE) cells were gifted from Marsico Lung Institute/Cystic Fibrosis Center at the University of North Carolina, Chapel Hill (Chapel Hill, NC) and were cultured as previously described^75^. Cells at passage 2 were transferred to microporous polyester inserts (0.4 mm pore size, Transwell-Clear; Corning Costar, Corning, NY) and fed with a 1:1 mixture of BEBM and Dulbecco’s Modification of Eagle’s Media (DMEM; Mediatech,Herndon, VA) supplemented with the same components detailed above and as previously described. Media was applied apically and basally until the cells were confluent and then basally after an air–liquid interface (ALI) was established. Cells were cultured at ALI for 14 days to promote relatively stable expression of goblet and ciliated cells before exposure to e-cig chemicals or mixture. Diacetyl and 2,3-pentanedione were diluted into culture medium, then mature, well-differentiated monolayers of cells were then exposed to control (medium), diacetyl (Sigma), or 2,3-pentanedione (Sigma) on the apical side for 24 h (n = 3 subjects, each treatment was performed in duplicate). Total RNA samples for RNA-Seq were isolated using miRNeasy kit (Qiagen).

### RNA-Seq Library Preparation and Sequencing

Polyadenylated mRNAs were selected from total RNA samples using oligo-dT-conjugated magnetic beads on an Apollo324 automated workstation (PrepX PolyA mRNA isolation kit, Takara Bio USA). Entire poly-adenylated RNA samples were immediately converted into stranded Illumina sequencing libraries using 200 bp fragmentation and sequential adapter addition on an Apollo324 automated workstation following manufacturer’s specifications (PrepX RNA-Seq for Illumina Library kit, Takara Bio USA). Libraries were enriched and indexed using 12 cycles of amplification (LongAmp Taq 2x MasterMix, New England BioLabs Inc.) with PCR primers which include a 6 bp index sequence to allow for multiplexing (custom oligo order from Integrated DNA Technologies). Excess PCR reagents were removed using magnetic bead-based cleanup on an Apollo324 automated workstation (PCR Clean DX beads, Aline Biosciences). Resulting libraries were assessed using a 2200 TapeStation (Agilent Technologies) and quantified by QPCR (Kapa Biosystems). Libraries were pooled and sequenced on one lane of a HiSeq2500 high output v3 flow cell using single end, 50 bp reads (Illumina).

### RNA-Seq Data Analysis

Taffeta scripts (https://github.com/blancahimes/taffeta) were used to analyze the RNA-Seq data, which included trimming of adapters using trimmomatic (v.0.32)^76^ and using FastQC (v.0.11.2) to obtain overall QC metrics. Trimmed reads for each sample were aligned with STAR (v. 2.5.2b) to the reference homo sapiens build 38 UCSC file (hg38) genome obtained from the Illumina, Inc. iGenomes resource^77^. Additional QC parameters were obtained to assess whether reads were appropriately mapped. Bamtools (v.2.3.0)^78^ was used to count/summarize the number of mapped reads, including junction spanning reads. The Picard Tools (v.1.96; http://picard.sourceforge.net) RnaSeqMetrics function was used to compute the number of bases assigned to various classes of RNA, according to the hg38 refFlat file available as a UCSC Genome Table. For each sample, HTSeq (v.0.6.1) was used to quantify genes based on reads that mapped to the provided hg38 reference files^79^. The DESeq2R package (v. 1.12.4) was used to measure significance of differentially expressed genes between the exposed (N = 4) and control (N = 4) samples and create plots of the results^80^. The reported adjusted p-values are false-discovery rate corrected to 5% according to the procedure in DESeq2 that accounts for the large number of comparisons made. An adjusted p-value < 0.05 was considered significant. The NIH Database for Annotation, Visualization and Integrated Discovery (DAVID) was used to perform gene functional annotation clustering using Homo Sapiens as background, and default options and annotation categories (Disease: OMIM_DISEASE; Functional Categories: COG_ONTOLOGY, SP_PIR_KEYWORDS, UP_SEQ_FEATURE; Gene_Ontology: GOTERM_BP_FAT, GOTERM_CC_FAT, GOTERM_MF_FAT; Pathway: BBID, BIOCARTA, KEGG_PATHWAY; Protein_Domains: INTERPRO, PIR_SUPERFAMILY, SMART)^81^.

### qPT-PCR validation

RNA was reverse transcribed using iScript cDNA Synthesis kit (Biorad). The resulting cDNA was amplified using 2x SYBR mix (Qiagen) and 1 μM of each primer in a StepOne Plus Thermocycler (Applied Biosystems) in Quantitative Reverse Transcriptase Polymerase Chain Reaction (qRT-PCR). Melt curves were checked for single-length amplification products. Fold changes were calculated using the 2-ΔΔCt method^82^. GAPDH was the housekeeping gene used for normalization in all qPCR assays. All primers used in this study and their respective sources or design are listed in Supplementary Table S7.

### Immunofluorescence staining

Immunofluorescence staining on ALI-cultured NHBE cells was performed as described previously with some modifications^83^. First, cells were fixed using 4% paraformaldehyde. Then, cells were blocked with PBS supplemented with 5% BSA and 0.2% Triton X-100 for 1 hour at room temperature. Primary antibody incubation was performed overnight at 4C in PBS supplemented with 1% BSA and 0.2% Triton X-100 using anti-β-tubulin IV (Sigma) or anti-MUC5AC (Thermo) at 1:100 dilution. Secondary antibodies conjugated with Alexa-fluor 488 (Life Technologies) were used at 1: 100 dilution. 4′-6-Diamidino-2-phenylindole, dihydrochloride was used to label the nuclear DNA and samples were mounted with Vectashield antifade mounting medium (Vector Labs, Burlingame, Calif). Confocal images were taken using Zeiss AxioObserver Z1 or Leica STP8000 and processed using ImageJ.

### Statistical analysis

Statistical analysis was performed with SigmaStat 4.0 (San Jose, CA 95131, USA). Data were analyzed by either t-test or one-way analysis of variance (ANOVA). If significant effects were detected, the ANOVA was followed by Holm-Sidak post hoc comparison of means. A P < 0.05 was considered statistically different. Data were expressed as means ± SEM.

## Supplementary information

Supplementary information
