# Supplementary material for: Transcriptomic response of primary human airway epithelial cells to flavoring chemicals in electronic cigarettes
Source: Sci Rep. 2019 Feb 1;9:1400. doi: 10.1038/s41598-018-37913-9 (PMC6358614; doi:10.1038/s41598-018-37913-9)
Supplement: Supplementary file 1 — Supplementary information [file 41598_2018_37913_MOESM1_ESM.docx]

**Transcriptomic response of primary human airway epithelial cells to flavoring chemicals in electronic cigarettes**

Hae-Ryung Park^1^, Michael O’Sullivan^1^, Jose Vallarino^1^, Maya Shumyatcher^2^, Blanca E. Himes^2^, Jin-Ah Park^1^, David C. Christiani^1^, Joseph Allen^1,*^, and Quan Lu^1,3*^

^1^Department of Environmental Health, Harvard T.H. Chan School of Public Health, Boston, Massachusetts 02115

^2^Department of Biostatistics, Epidemiology and Informatics, University of Pennsylvania, Philadelphia, Pennsylvania 19104

^3^Department of Genetics and Complex Diseases, Harvard T.H. Chan School of Public Health, Boston, Massachusetts 02115

**Supplementary Figure 1**. Cytotoxicity of diacetyl and 2,3-pentanedione in NHBE cells.

**Supplementary Table 1**. Differentially regulated genes with 25 ppm diacetyl treatment in NHBE cells.

| Diacetyl vs control | | |
| --- | --- | --- |
| Gene | Fold Change | Padj |
| *PAPSS2* | 0.51 | 2.68E-25 |
| *CDC20B* | 0.56 | 1.01E-16 |
| *MIR205HG* | 0.62 | 2.13E-08 |
| *MACROD2* | 0.62 | 0.0002 |
| *SORCS2* | 0.62 | 5.79E-07 |
| *TAX1BP3* | 1.48 | 0.0018 |
| *DHRS9* | 1.52 | 4.04E-10 |
| *SNHG14* | 1.52 | 4.16E-06 |
| *KRT13* | 1.59 | 4.87E-08 |
| *KRT4* | 1.80 | 2.70E-19 |

**Supplementary Table 2**. Differentially regulated genes with 100 ppm 2,3-pentanedione treatment in NHBE cells.

| 2,3-Pentanedionel vs control | | |
| --- | --- | --- |
| Gene | Fold Change | Padj |
| *CSF3* | 0.24 | 1.46E-15 |
| *IL33* | 0.25 | 2.44E-48 |
| *PAPSS2* | 0.29 | 1.16E-81 |
| *PTGS2* | 0.30 | 1.15E-48 |
| *CPXM2* | 0.32 | 4.20E-12 |
| *KRT14* | 2.14 | 8.12E-10 |
| *SCGB1A1* | 2.21 | 1.14E-24 |
| *KRT16* | 2.44 | 4.78E-11 |
| *GPX2* | 2.60 | 1.65E-13 |
| *KRT4* | 2.96 | 2.10E-72 |

**Supplementary Table 3**. Enriched terms in the gene list differentially regulated by diacetyl.

| **Annotation Cluster 1** | **Enrichment Score: 4.600** | | | |
| --- | --- | --- | --- | --- |
| Category | Term | Genes | Fold Enrichment | Benjamini |
| UP_KEYWORDS | Cell projection | CFAP126, S100P, DNAH12, DRC3, B9D1, WASF2, DNAH3, CFAP221, SPAG17, EPHA2, CFAP52, DNAH6, CFAP70, PROM1, TENM4, CFAP43, DNAI1, TEKT1, DNAAF1, CC2D2A | 3.683 | 4.60E-04 |
| UP_KEYWORDS | Cilium | CFAP70, PROM1, DNAH12, DNAI1, TEKT1, DNAAF1, CC2D2A, DNAH3, CFAP221, SPAG17, DNAH6 | 7.056 | 4.34E-04 |
| UP_KEYWORDS | Cytoskeleton | PPP4R3A, CFAP126, DNAH12, DRC3, B9D1, WASF2, DNAH3, CFAP221, CCDC146, SPAG17, FLNA, DNAH6, DNAI1, TEKT1, DMD, DNAAF1, CC2D2A, CCDC78, SEPT9 | 2.217 | 0.062 |
| **Annotation Cluster 2** | **Enrichment Score: 3.402** | | | |
| Category | Term | Genes | Fold Enrichment | Benjamini |
| INTERPRO | IPR018039:Intermediate filament protein, conserved site | KRT19, KRT5, KRT15, KRT8, KRT14, KRT13, KRT4 | 14.195 | 0.003 |
| UP_SEQ_FEATURE | region of interest:Coil 2 | KRT19, KRT5, KRT15, KRT8, KRT14, KRT13, KRT4 | 13.325 | 0.011 |
| UP_SEQ_FEATURE | region of interest:Linker 12 | KRT19, KRT5, KRT15, KRT8, KRT14, KRT13, KRT4 | 13.325 | 0.011 |
| UP_SEQ_FEATURE | region of interest:Linker 1 | KRT19, KRT5, KRT15, KRT8, KRT14, KRT13, KRT4 | 12.244 | 0.009 |
| UP_SEQ_FEATURE | region of interest:Coil 1A | KRT19, KRT5, KRT15, KRT8, KRT14, KRT13, KRT4 | 12.244 | 0.009 |
| UP_SEQ_FEATURE | region of interest:Coil 1B | KRT19, KRT5, KRT15, KRT8, KRT14, KRT13, KRT4 | 12.244 | 0.009 |
| UP_SEQ_FEATURE | region of interest:Rod | KRT19, KRT5, KRT15, KRT8, KRT14, KRT13, KRT4 | 12.081 | 0.006 |
| UP_KEYWORDS | Intermediate filament | KRT19, KRT5, KRT15, KRT8, KRT14, KRT13, KRT4 | 12.071 | 0.002 |
| UP_SEQ_FEATURE | region of interest:Head | KRT19, KRT5, KRT15, KRT8, KRT14, KRT13, KRT4 | 11.767 | 0.005 |
| INTERPRO | IPR001664:Intermediate filament protein | KRT19, KRT5, KRT15, KRT8, KRT14, KRT13, KRT4 | 11.647 | 0.005 |
| SMART | SM01391:SM01391 | KRT19, KRT5, KRT15, KRT8, KRT14, KRT13, KRT4 | 10.915 | 0.004 |
| INTERPRO | IPR009053:Prefoldin | KRT19, KRT15, KRT8, KRT14, LAMB1 | 24.958 | 0.005 |
| UP_SEQ_FEATURE | site:Stutter | KRT19, KRT5, KRT8, KRT14, KRT4 | 17.978 | 0.025 |
| GOTERM_CC_DIRECT | GO:0005882~intermediate filament | KRT19, KRT5, KRT15, KRT8, KRT14, KRT13, KRT4 | 8.007 | 0.012 |
| UP_SEQ_FEATURE | region of interest:Tail | KRT5, KRT15, KRT8, KRT14, KRT13, KRT4 | 9.831 | 0.045 |
| GOTERM_MF_DIRECT | GO:0005198~structural molecule activity | LAMA2, KRT19, KRT5, KRT15, KRT8, KRT14, KRT13, KRT4, LAMB1 | 4.768 | 0.139 |
| UP_KEYWORDS | Keratin | KRT19, KRT5, KRT15, KRT8, KRT14, KRT13, KRT4 | 6.075 | 0.040 |
| INTERPRO | IPR002957:Keratin, type I | KRT19, KRT15, KRT14, KRT13 | 15.731 | 0.162 |
| GOTERM_CC_DIRECT | GO:0045095~keratin filament | KRT5, KRT8, KRT14, KRT13, KRT4 | 6.462 | 0.132 |
| GOTERM_MF_DIRECT | GO:0005200~structural constituent of cytoskeleton | KRT19, KRT5, DMD, KRT15, KRT14 | 5.948 | 0.463 |
| INTERPRO | IPR003054:Type II keratin | KRT5, KRT8, KRT4 | 13.905 | 0.531 |
| GOTERM_BP_DIRECT | GO:0007010~cytoskeleton organization | KRT5, DMD, KRT15, KRT13, KRT4 | 4.106 | 0.858 |
| GOTERM_MF_DIRECT | GO:0097110~scaffold protein binding | KRT5, KRT15, KRT8 | 8.179 | 0.850 |
| UP_SEQ_FEATURE | compositionally biased region:Gly-rich | KRT5, KRT15, KRT13, KRT4 | 2.055 | 1.000 |
| UP_SEQ_FEATURE | compositionally biased region:Ser-rich | KIAA1522, KRT5, KRT8, KRT4, TPBG | 1.468 | 1.000 |
| **Annotation Cluster 3** | **Enrichment Score: 2.321** | | | |
| Category | Term | Genes | Fold Enrichment | Benjamini |
| UP_KEYWORDS | Cilium biogenesis/degradation | CFAP126, DNAI1, B9D1, CC2D2A, CFAP221, FLNA, SPAG17, CCDC78 | 7.177 | 0.007 |
| GOTERM_BP_DIRECT | GO:0060271~cilium morphogenesis | NOTCH1, B9D1, DNAAF1, CC2D2A, CFAP221, FLNA | 5.833 | 0.467 |
| GOTERM_BP_DIRECT | GO:0042384~cilium assembly | B9D1, CC2D2A, FLNA | 3.199 | 0.998 |

**Supplementary Table 4**. Enriched terms in the gene list differentially regulated by 2,3-pentanedione.

| **Annotation Cluster 1** | **Enrichment Score: 12.392** | | | |
| --- | --- | --- | --- | --- |
| Category | Term | Genes | Fold Enrichment | Benjamini |
| GOTERM_CC_DIRECT | GO:0005913~cell-cell adherens junction | ALDOA, CHMP4B, LMO7, GIPC1, ANLN, SFN, GPRC5A, PRDX1, PKM, PPL, BAG3, RAB11B, FAM129B, ZYX, EHD1, ENO1, ARGLU1, DAB2IP, BSG, S100P, LAD1, TRIM29, ANXA1, SLC3A2, EEF2, MYH9, SLC9A3R2, EPHA2, FLNA, JUP, EIF4G1, CCNB2, EVPL, SCYL1, CGN, PKP3, SPTBN1, SPTAN1, SEPT9, SH3GL1 | 4.315 | 6.88E-12 |
| GOTERM_MF_DIRECT | GO:0098641~cadherin binding involved in cell-cell adhesion | ALDOA, CHMP4B, GIPC1, ANLN, SFN, GPRC5A, PRDX1, PKM, PPL, BAG3, RAB11B, FAM129B, EHD1, ENO1, ARGLU1, DAB2IP, BSG, S100P, LAD1, TRIM29, ANXA1, SLC3A2, EEF2, MYH9, SLC9A3R2, FLNA, EPHA2, JUP, EIF4G1, CCNB2, EVPL, SCYL1, CGN, PKP3, SPTBN1, SPTAN1, SEPT9, SH3GL1 | 4.372 | 6.12E-11 |
| GOTERM_BP_DIRECT | GO:0098609~cell-cell adhesion | ALDOA, CHMP4B, GIPC1, ANLN, SFN, GPRC5A, PRDX1, PKM, PPL, BAG3, RAB11B, FAM129B, EHD1, ENO1, ARGLU1, DAB2IP, BSG, S100P, LAD1, TRIM29, SLC3A2, EEF2, SLC9A3R2, EPHA2, EIF4G1, CCNB2, EVPL, SCYL1, CGN, SPTBN1, SEPT9, SH3GL1, SPTAN1 | 4.106 | 6.69E-08 |
| **Annotation Cluster 2** | **Enrichment Score: 5.127** | | | |
| Category | Term | Genes | Fold Enrichment | Benjamini |
| UP_KEYWORDS | Ciliopathy | DNAH11, CEP120, IFT80, CENPF, DNAI2, ARL6, DNAH5, TMEM67, WDR19, DNAI1, DNAAF1, DYNC2H1, TCTN1, DNAL1, IFT140, NPHP1 | 4.743 | 4.82E-05 |
| GOTERM_BP_DIRECT | GO:0060271~cilium morphogenesis | IFT80, STK36, CFAP221, ARL6, DNAH5, FLNA, TTLL3, TMEM67, NOTCH1, WDR19, DNAAF1, DYNC2H1, TCTN1, EHD1, ATP6V0D1, TTC21A, IFT140 | 4.215 | 0.003 |
| GOTERM_BP_DIRECT | GO:0042384~cilium assembly | IFT80, STK36, ABLIM3, DNAI2, ARL6, FLNA, DNAH5, TTLL3, TMEM67, WDR19, DYNC2H1, ABCC4, ATP6V0D1, EHD1, IFT140 | 4.079 | 0.012 |
| UP_KEYWORDS | Cilium biogenesis/degradation | STK36, CFAP221, DNAI2, ARL6, FLNA, SPAG17, TMEM67, WDR19, DNAI1, DYNC2H1, TCTN1, EHD1, ATP6V0D1, IFT140, NPHP1 | 3.786 | 0.001 |
| **Annotation Cluster 3** | **Enrichment Score: 4.643** | | | |
| Category | Term | Genes | Fold Enrichment | Benjamini |
| UP_SEQ_FEATURE | region of interest:AAA 1 | DNAH11, DNAH12, DYNC2H1, DNAH3, DNAH1, DNAH7, DNAH5, DNAH6 | 20.921 | 5.77E-05 |
| UP_SEQ_FEATURE | region of interest:AAA 3 | DNAH11, DNAH12, DYNC2H1, DNAH3, DNAH1, DNAH7, DNAH5, DNAH6 | 20.921 | 5.77E-05 |
| UP_SEQ_FEATURE | region of interest:AAA 2 | DNAH11, DNAH12, DYNC2H1, DNAH3, DNAH1, DNAH7, DNAH5, DNAH6 | 20.921 | 5.77E-05 |
| UP_SEQ_FEATURE | region of interest:AAA 5 | DNAH11, DNAH12, DYNC2H1, DNAH3, DNAH1, DNAH7, DNAH5, DNAH6 | 20.921 | 5.77E-05 |
| UP_SEQ_FEATURE | region of interest:AAA 4 | DNAH11, DNAH12, DYNC2H1, DNAH3, DNAH1, DNAH7, DNAH5, DNAH6 | 20.921 | 5.77E-05 |
| UP_SEQ_FEATURE | region of interest:Stem | DNAH11, DNAH12, DYNC2H1, DNAH3, DNAH1, DNAH7, DNAH5, DNAH6 | 20.921 | 5.77E-05 |
| UP_SEQ_FEATURE | region of interest:Stalk | DNAH11, DNAH12, DYNC2H1, DNAH3, DNAH1, DNAH7, DNAH5, DNAH6 | 20.921 | 5.77E-05 |
| INTERPRO | IPR024317:Dynein heavy chain, P-loop containing D4 domain | DNAH11, DNAH12, DYNC2H1, DNAH3, DNAH1, DNAH7, DNAH5, DNAH6 | 18.890 | 7.80E-05 |
| INTERPRO | IPR004273:Dynein heavy chain domain | DNAH11, DNAH12, DYNC2H1, DNAH3, DNAH1, DNAH7, DNAH5, DNAH6 | 17.709 | 6.76E-05 |
| INTERPRO | IPR013602:Dynein heavy chain, domain-2 | DNAH11, DNAH12, DYNC2H1, DNAH3, DNAH1, DNAH7, DNAH5, DNAH6 | 17.709 | 6.76E-05 |
| INTERPRO | IPR026983:Dynein heavy chain | DNAH11, DNAH12, DYNC2H1, DNAH3, DNAH1, DNAH7, DNAH5, DNAH6 | 17.709 | 6.76E-05 |
| INTERPRO | IPR024743:Dynein heavy chain, coiled coil stalk | DNAH11, DNAH12, DYNC2H1, DNAH3, DNAH1, DNAH7, DNAH5, DNAH6 | 17.709 | 6.76E-05 |
| UP_KEYWORDS | Dynein | DNAH11, DNAH12, DNAI1, DYNC2H1, DNAH3, DNAH1, DNAI2, DNAH7, DNAH5, DNAH6 | 11.319 | 6.47E-06 |
| UP_SEQ_FEATURE | region of interest:AAA 6 | DNAH11, DYNC2H1, DNAH3, DNAH1, DNAH7, DNAH5, DNAH6 | 19.714 | 3.58E-04 |
| COG_ONTOLOGY | Cytoskeleton | DNAH11, DNAH12, DYNC2H1, DNAH3, DNAH1, DNAH7, DNAH5, DNAH6 | 9.506 | 1.96E-04 |
| INTERPRO | IPR011704:ATPase, dynein-related, AAA domain | DNAH11, DNAH12, DYNC2H1, DNAH1, DNAH5, DNAH6 | 15.179 | 0.006 |
| UP_KEYWORDS | Motor protein | MYL6, DNAH11, DNAH12, DNAH3, DNAH1, DNAI2, DNAH7, MYH9, DNAH5, DNAH6, KIF1C, DNAI1, DYNC2H1, MYH14 | 3.874 | 0.002 |
| GOTERM_CC_DIRECT | GO:0005858~axonemal dynein complex | DNAH3, DNAH1, DNAI2, DNAH7, DNAH6 | 17.423 | 0.005 |
| GOTERM_MF_DIRECT | GO:0003777~microtubule motor activity | KIF1C, DNAH11, DNAH12, DYNC2H1, DNAH3, DNAH1, DNAI2, DNAH7, DNAH5, DNAH6 | 4.170 | 0.048 |
| GOTERM_MF_DIRECT | GO:0016887~ATPase activity | DNAH11, ATP5D, DNAH12, CLU, DNAH1, MYH9, DNAH7, ATP13A2, DNAH5, DNAH6, ABCA5, KIF1C, PSMC5, DYNC2H1, SMARCA1 | 2.735 | 0.084 |
| KEGG_PATHWAY | hsa05016:Huntington's disease | DNAH11, ATP5D, CLTB, DNAH12, AP2S1, DNAH3, DNAH1, DNAI2, DNAH7, DNAH5, DNAH6, POLR2A, SOD2, DNAI1, CREB3L1, DNAL1 | 2.379 | 0.277 |
| GOTERM_BP_DIRECT | GO:0060285~cilium-dependent cell motility | CFAP44, DNAH3, DNAH1, DNAH7 | 12.261 | 0.316 |
| UP_KEYWORDS | Microtubule | DNAH11, DNAH12, NEK2, RUSC1, DNAH3, DNAH1, DNAI2, DNAH7, SPAG17, DNAH5, TTLL3, DNAH6, KIF1C, DNAI1, DYNC2H1, TUBA1A, NICN1 | 2.268 | 0.050 |
| INTERPRO | IPR003593:AAA+ ATPase domain | DNAH11, DNAH12, PSMC5, DYNC2H1, DNAH3, ABCC4, DNAH7, DNAH5, AK9, ABCA5, DNAH6 | 2.865 | 0.353 |
| SMART | SM00382:AAA | DNAH11, DNAH12, PSMC5, DYNC2H1, DNAH3, ABCC4, DNAH7, DNAH5, AK9, ABCA5, DNAH6 | 2.658 | 0.503 |
| GOTERM_CC_DIRECT | GO:0005874~microtubule | DNAH11, DNAH12, NEK2, RUSC1, DNAH3, DNAH1, DNAI2, DNAH7, SPAG17, DNAH5, TTLL3, DNAH6, KIF1C, DNAI1, DYNC2H1, TUBA1A, NICN1, SEPT9 | 2.017 | 0.154 |
| GOTERM_CC_DIRECT | GO:0030286~dynein complex | DNAH11, DNAH12, DYNC2H1, DNAH1 | 6.335 | 0.277 |
| INTERPRO | IPR013594:Dynein heavy chain, domain-1 | DNAH11, DYNC2H1, DNAH5 | 11.806 | 0.577 |
| GOTERM_BP_DIRECT | GO:0007018~microtubule-based movement | KIF1C, DNAH12, AP2S1, DYNC2H1, DNAH3, DNAH5, DNAH6 | 2.914 | 0.736 |
| GOTERM_BP_DIRECT | GO:0030317~sperm motility | DNAH11, DNAI1, DNAH1, DNAH5 | 2.498 | 0.977 |
| BIOCARTA | h_Lis1Pathway:Lissencephaly gene (LIS1) in neuronal migration and development | DNAH3, DNAH1, DNAH6 | 3.129 | 0.992 |
| **Annotation Cluster 4** | **Enrichment Score: 4.2010** | | | |
| Category | Term | Genes | Fold Enrichment | Benjamini |
| GOTERM_MF_DIRECT | GO:0005198~structural molecule activity | CLTB, CLDN4, VIM, CLDN10, CLDN12, KRT80, KRT5, KRT7, KRT8, UPK1B, KRT4, LAMB1, TUBA1A, NPHP1, LAD1, ANXA1, LMNA, KRT13, JUP, KRT19, EVPL, LAMA3, KRT16, KRT15, KRT14, SPRR3, MAP7D1 | 3.647 | 9.79E-06 |
| UP_SEQ_FEATURE | region of interest:Head | VIM, LMNA, KRT13, KRT19, KRT80, KRT5, KRT16, CGN, KRT7, KRT15, KRT8, KRT14, KRT4 | 6.181 | 5.04E-04 |
| UP_SEQ_FEATURE | region of interest:Coil 2 | KRT19, KRT80, KRT5, KRT16, KRT15, KRT7, KRT8, VIM, KRT14, LMNA, KRT13, KRT4 | 6.461 | 7.74E-04 |
| UP_SEQ_FEATURE | region of interest:Coil 1A | KRT19, KRT80, KRT5, KRT16, KRT15, KRT7, KRT8, VIM, KRT14, LMNA, KRT13, KRT4 | 5.937 | 0.002 |
| UP_SEQ_FEATURE | region of interest:Coil 1B | KRT19, KRT80, KRT5, KRT16, KRT15, KRT7, KRT8, VIM, KRT14, LMNA, KRT13, KRT4 | 5.937 | 0.002 |
| UP_SEQ_FEATURE | region of interest:Linker 1 | KRT19, KRT80, KRT5, KRT16, KRT15, KRT7, KRT8, VIM, KRT14, LMNA, KRT13, KRT4 | 5.937 | 0.002 |
| UP_SEQ_FEATURE | region of interest:Rod | KRT19, KRT80, KRT5, KRT16, KRT15, KRT7, KRT8, VIM, KRT14, LMNA, KRT13, KRT4 | 5.858 | 0.001 |
| UP_KEYWORDS | Intermediate filament | KRT19, KRT80, KRT5, KRT16, KRT15, KRT7, KRT8, VIM, KRT14, LMNA, KRT13, KRT4 | 5.821 | 1.79E-04 |
| UP_SEQ_FEATURE | region of interest:Tail | KRT80, KRT5, CGN, KRT16, KRT15, KRT7, KRT8, VIM, KRT14, LMNA, KRT13, KRT4 | 5.561 | 0.002 |
| INTERPRO | IPR018039:Intermediate filament protein, conserved site | KRT19, KRT5, KRT16, KRT15, KRT7, KRT8, VIM, KRT14, LMNA, KRT13, KRT4 | 6.087 | 0.004 |
| INTERPRO | IPR001664:Intermediate filament protein | KRT19, KRT80, KRT5, KRT16, KRT15, KRT7, KRT8, VIM, KRT14, LMNA, KRT13, KRT4 | 5.449 | 0.003 |
| SMART | SM01391:SM01391 | KRT19, KRT80, KRT5, KRT16, KRT15, KRT7, KRT8, VIM, KRT14, LMNA, KRT13, KRT4 | 5.259 | 0.004 |
| UP_SEQ_FEATURE | region of interest:Linker 12 | KRT19, KRT80, KRT5, KRT16, KRT15, KRT7, KRT8, VIM, KRT14, KRT13, KRT4 | 5.922 | 0.002 |
| UP_SEQ_FEATURE | site:Stutter | KRT19, KRT80, KRT5, KRT7, KRT8, KRT14, LMNA, KRT4 | 8.136 | 0.006 |
| GOTERM_CC_DIRECT | GO:0005882~intermediate filament | VIM, LMNA, KRT13, JUP, KRT19, KRT80, KRT5, KRT16, KRT7, KRT15, KRT8, KRT14, KRT4 | 4.009 | 0.004 |
| GOTERM_MF_DIRECT | GO:0005200~structural constituent of cytoskeleton | ACTB, KRT19, KRT5, KRT16, DMD, PPL, KRT15, VIM, KRT14, SPTBN1, TUBA1A, SPTAN1 | 3.639 | 0.039 |
| INTERPRO | IPR009053:Prefoldin | KRT19, KRT15, KRT7, KRT8, KRT14, LAMB1 | 8.173 | 0.074 |
| INTERPRO | IPR003054:Type II keratin | KRT80, KRT5, KRT7, KRT8, KRT4 | 6.325 | 0.419 |
| INTERPRO | IPR002957:Keratin, type I | KRT19, KRT16, KRT15, KRT14, KRT13 | 5.366 | 0.443 |
| UP_KEYWORDS | Keratin | KRT19, KRT80, KRT5, KRT16, KRT15, KRT7, KRT8, KRT14, KRT13, KRT4 | 2.441 | 0.184 |
| GOTERM_CC_DIRECT | GO:0045095~keratin filament | KRT80, KRT5, KRT7, KRT8, KRT14, KRT13, KRT4 | 2.439 | 0.431 |
| GOTERM_MF_DIRECT | GO:0097110~scaffold protein binding | KRT5, KRT15, KRT8, VIM | 2.780 | 0.909 |
| **Annotation Cluster 5** | **Enrichment Score: 3.795** | | | |
| Category | Term | Genes | Fold Enrichment | Benjamini |
| UP_SEQ_FEATURE | repeat:WD 5 | IFT80, STRN4, LRBA, CDC20B, NBEA, BOP1, DNAI2, LLGL2, CFAP52, PRPF19, WSB1, CFAP44, WDR19, CFAP43, DNAI1, GNB2, WDR78, GRWD1, AAMP, ATG16L2, IFT140 | 3.244 | 0.002 |
| UP_KEYWORDS | WD repeat | PAN2, IFT80, STRN4, LRBA, CDC20B, NBEA, BOP1, DNAI2, LLGL2, CFAP52, PRPF19, WSB1, CFAP44, WDR19, CFAP43, DNAI1, GNB2, WDR78, GRWD1, AAMP, ATG16L2, IFT140 | 2.924 | 0.001 |
| UP_SEQ_FEATURE | repeat:WD 4 | IFT80, STRN4, LRBA, CDC20B, NBEA, BOP1, DNAI2, LLGL2, CFAP52, PRPF19, WSB1, CFAP44, WDR19, CFAP43, DNAI1, GNB2, WDR78, GRWD1, AAMP, ATG16L2, IFT140 | 3.015 | 0.004 |
| UP_SEQ_FEATURE | repeat:WD 3 | IFT80, STRN4, LRBA, CDC20B, NBEA, BOP1, DNAI2, LLGL2, CFAP52, PRPF19, WSB1, CFAP44, WDR19, CFAP43, DNAI1, GNB2, WDR78, GRWD1, AAMP, ATG16L2, IFT140 | 2.858 | 0.007 |
| UP_SEQ_FEATURE | repeat:WD 2 | IFT80, STRN4, LRBA, CDC20B, NBEA, BOP1, DNAI2, LLGL2, CFAP52, PRPF19, WSB1, CFAP44, WDR19, CFAP43, DNAI1, GNB2, WDR78, GRWD1, AAMP, ATG16L2, IFT140 | 2.766 | 0.010 |
| UP_SEQ_FEATURE | repeat:WD 1 | IFT80, STRN4, LRBA, CDC20B, NBEA, BOP1, DNAI2, LLGL2, CFAP52, PRPF19, WSB1, CFAP44, WDR19, CFAP43, DNAI1, GNB2, WDR78, GRWD1, AAMP, ATG16L2, IFT140 | 2.766 | 0.010 |
| INTERPRO | IPR017986:WD40-repeat-containing domain | PAN2, IFT80, STRN4, LRBA, CDC20B, NBEA, BOP1, DNAI2, LLGL2, CFAP52, PRPF19, WSB1, CFAP44, WDR19, CFAP43, DNAI1, GNB2, WDR78, GRWD1, AAMP, ATG16L2, IFT140 | 2.546 | 0.026 |
| INTERPRO | IPR015943:WD40/YVTN repeat-like-containing domain | PAN2, IFT80, PLXNB2, STRN4, LRBA, CDC20B, NBEA, BOP1, DNAI2, LLGL2, CFAP52, PRPF19, WSB1, CFAP44, WDR19, CFAP43, DNAI1, GNB2, WDR78, GRWD1, AAMP, ATG16L2, IFT140 | 2.461 | 0.025 |
| INTERPRO | IPR001680:WD40 repeat | IFT80, STRN4, LRBA, CDC20B, NBEA, BOP1, DNAI2, LLGL2, CFAP52, WSB1, PRPF19, CFAP44, WDR19, DNAI1, GNB2, WDR78, GRWD1, AAMP, ATG16L2, IFT140 | 2.643 | 0.027 |
| UP_SEQ_FEATURE | repeat:WD 6 | IFT80, STRN4, LRBA, CDC20B, BOP1, LLGL2, CFAP52, WSB1, PRPF19, CFAP44, CFAP43, WDR19, GNB2, WDR78, AAMP, ATG16L2 | 2.989 | 0.035 |
| SMART | SM00320:WD40 | IFT80, STRN4, LRBA, CDC20B, NBEA, BOP1, DNAI2, LLGL2, CFAP52, WSB1, PRPF19, CFAP44, WDR19, DNAI1, GNB2, WDR78, GRWD1, AAMP, ATG16L2, IFT140 | 2.462 | 0.060 |
| UP_SEQ_FEATURE | repeat:WD 7 | PRPF19, CFAP44, CFAP43, IFT80, GNB2, STRN4, AAMP, CDC20B, BOP1, ATG16L2, LLGL2, CFAP52 | 2.910 | 0.235 |
| INTERPRO | IPR019775:WD40 repeat, conserved site | WSB1, PRPF19, CFAP44, GNB2, GRWD1, STRN4, AAMP, CDC20B, BOP1, ATG16L2, LLGL2, CFAP52 | 2.560 | 0.401 |
| **Annotation Cluster 6** | **Enrichment Score: 3.147** | | | |
| Category | Term | Genes | Fold Enrichment | Benjamini |
| GOTERM_BP_DIRECT | GO:0001525~angiogenesis | DAB2IP, FLT1, PTGS2, CXCL8, ANPEP, MYH9, NCL, KDR, PRKD2, GPI, PDCL3, CLIC4, ID1, VEGFA, PLCD3, AAMP, ERAP1, TNFAIP2, ANGPTL4 | 2.873 | 0.043 |
| UP_KEYWORDS | Angiogenesis | PRKD2, GPI, DAB2IP, NOTCH1, PDCL3, FLT1, VEGFA, AAMP, ANPEP, TNFAIP2, EPHA2, KDR, ANGPTL4 | 3.854 | 0.003 |
| GOTERM_BP_DIRECT | GO:0045766~positive regulation of angiogenesis | PRKD2, PDCL3, FLT1, VEGFA, CXCL8, HSPB1, ERAP1, KDR, ANGPTL4 | 2.639 | 0.669 |
| **Annotation Cluster 7** | **Enrichment Score: 2.708** | | | |
| Category | Term | Genes | Fold Enrichment | Benjamini |
| GOTERM_BP_DIRECT | GO:0003341~cilium movement | DNAH11, DNAI1, DNAAF1, CFAP221, DNAH1, DNAI2, DNAH7, DNAH5 | 9.991 | 0.009 |
| UP_KEYWORDS | Primary ciliary dyskinesia | DNAH11, DNAI1, DNAAF1, CENPF, DNAI2, DNAL1, DNAH5 | 8.171 | 0.003 |
| UP_KEYWORDS | Kartagener syndrome | DNAH11, DNAI1, DNAH5 | 37.352 | 0.032 |
| GOTERM_CC_DIRECT | GO:0036157~outer dynein arm | DNAI1, DNAI2, DNAH5 | 20.907 | 0.146 |
| GOTERM_BP_DIRECT | GO:0036158~outer dynein arm assembly | DNAI1, DNAAF1, DNAI2, DNAH5 | 8.992 | 0.493 |
| GOTERM_BP_DIRECT | GO:0030317~sperm motility | DNAH11, DNAI1, DNAH1, DNAH5 | 2.498 | 0.977 |

**Supplementary Table 5**. Enriched terms in the gene list differentially regulated by both diacetyl and 2,3-pentanedione.

| **Annotation Cluster 1** | **Enrichment Score: 3.512** | | | |
| --- | --- | --- | --- | --- |
| Category | Term | Genes | Fold Enrichment | Benjamini |
| INTERPRO | IPR018039:Intermediate filament protein, conserved site | KRT19, KRT5, KRT15, KRT8, KRT14, KRT13, KRT4 | 16.370 | 0.001 |
| UP_SEQ_FEATURE | region of interest:Coil 2 | KRT19, KRT5, KRT15, KRT8, KRT14, KRT13, KRT4 | 15.299 | 0.004 |
| UP_SEQ_FEATURE | region of interest:Linker 12 | KRT19, KRT5, KRT15, KRT8, KRT14, KRT13, KRT4 | 15.299 | 0.004 |
| UP_SEQ_FEATURE | region of interest:Coil 1B | KRT19, KRT5, KRT15, KRT8, KRT14, KRT13, KRT4 | 14.058 | 0.004 |
| UP_SEQ_FEATURE | region of interest:Coil 1A | KRT19, KRT5, KRT15, KRT8, KRT14, KRT13, KRT4 | 14.058 | 0.004 |
| UP_SEQ_FEATURE | region of interest:Linker 1 | KRT19, KRT5, KRT15, KRT8, KRT14, KRT13, KRT4 | 14.058 | 0.004 |
| UP_SEQ_FEATURE | region of interest:Rod | KRT19, KRT5, KRT15, KRT8, KRT14, KRT13, KRT4 | 13.871 | 0.003 |
| UP_KEYWORDS | Intermediate filament | KRT19, KRT5, KRT15, KRT8, KRT14, KRT13, KRT4 | 13.859 | 0.001 |
| UP_SEQ_FEATURE | region of interest:Head | KRT19, KRT5, KRT15, KRT8, KRT14, KRT13, KRT4 | 13.510 | 0.002 |
| INTERPRO | IPR001664:Intermediate filament protein | KRT19, KRT5, KRT15, KRT8, KRT14, KRT13, KRT4 | 13.432 | 0.002 |
| SMART | SM01391:SM01391 | KRT19, KRT5, KRT15, KRT8, KRT14, KRT13, KRT4 | 12.515 | 0.001 |
| INTERPRO | IPR009053:Prefoldin | KRT19, KRT15, KRT8, KRT14, LAMB1 | 28.783 | 0.003 |
| UP_SEQ_FEATURE | site:Stutter | KRT19, KRT5, KRT8, KRT14, KRT4 | 20.641 | 0.014 |
| GOTERM_CC_DIRECT | GO:0005882~intermediate filament | KRT19, KRT5, KRT15, KRT8, KRT14, KRT13, KRT4 | 9.253 | 0.007 |
| UP_SEQ_FEATURE | region of interest:Tail | KRT5, KRT15, KRT8, KRT14, KRT13, KRT4 | 11.287 | 0.022 |
| UP_KEYWORDS | Keratin | KRT19, KRT5, KRT15, KRT8, KRT14, KRT13, KRT4 | 6.975 | 0.022 |
| GOTERM_MF_DIRECT | GO:0005198~structural molecule activity | KRT19, KRT5, KRT15, KRT8, KRT14, KRT13, KRT4, LAMB1 | 4.839 | 0.265 |
| INTERPRO | IPR002957:Keratin, type I | KRT19, KRT15, KRT14, KRT13 | 18.142 | 0.098 |
| GOTERM_CC_DIRECT | GO:0045095~keratin filament | KRT5, KRT8, KRT14, KRT13, KRT4 | 7.469 | 0.133 |
| GOTERM_MF_DIRECT | GO:0005200~structural constituent of cytoskeleton | KRT19, KRT5, DMD, KRT15, KRT14 | 6.790 | 0.521 |
| INTERPRO | IPR003054:Type II keratin | KRT5, KRT8, KRT4 | 16.036 | 0.435 |
| GOTERM_BP_DIRECT | GO:0007010~cytoskeleton organization | KRT5, DMD, KRT15, KRT13, KRT4 | 4.784 | 0.813 |
| GOTERM_MF_DIRECT | GO:0097110~scaffold protein binding | KRT5, KRT15, KRT8 | 9.337 | 0.913 |
| UP_SEQ_FEATURE | compositionally biased region:Gly-rich | KRT5, KRT15, KRT13, KRT4 | 2.359 | 0.998 |
| UP_KEYWORDS | Methylation | KIAA1522, TF, KRT19, KRT8, KRT13, EPHX1, KRT4, C6ORF132 | 1.218 | 0.960 |
| UP_SEQ_FEATURE | compositionally biased region:Ser-rich | KIAA1522, KRT5, KRT8, KRT4 | 1.348 | 1.000 |
| **Annotation Cluster 2** | **Enrichment Score: 1.975** | | | |
| Category | Term | Genes | Fold Enrichment | Benjamini |
| GOTERM_CC_DIRECT | GO:0005615~extracellular space | MUC1, CXCL1, TF, PSAP, CPXM2, ANPEP, IL33, ABI3BP, PTHLH, PROM1, CEL, CPAMD8, VEGFA, C1RL, CSTB, SERPINB2, LTF, CEACAM6, SERPINB4, IL5RA, LAMB1, TNFAIP2 | 2.440 | 0.010 |
| UP_KEYWORDS | Secreted | MUC1, MATN2, CXCL1, TF, GLB1L, PSAP, CPXM2, IL33, ABI3BP, PTHLH, CEL, SDC1, CPAMD8, VEGFA, C1RL, SERPINB2, LTF, LAMB1, TLL1, IGFBP5 | 1.552 | 0.447 |
| GOTERM_CC_DIRECT | GO:0005576~extracellular region | CXCL1, TF, GLB1L, PSAP, IL33, FLNA, PTHLH, CEL, NOTCH1, CPAMD8, VEGFA, SERPINB2, LTF, LAMB1, TLL1, IGFBP5 | 1.484 | 0.705 |
| **Annotation Cluster 3** | **Enrichment Score: 1.891** | | | |
| Category | Term | Genes | Fold Enrichment | Benjamini |
| UP_KEYWORDS | Cilium | CFAP70, PROM1, DNAH12, DNAI1, TEKT1, DNAAF1, CC2D2A, DNAH3, CFAP221, SPAG17, DNAH6 | 8.101 | 2.34E-04 |
| UP_KEYWORDS | Cell projection | S100P, DNAH12, DRC3, WASF2, DNAH3, CFAP221, EPHA2, SPAG17, CFAP52, DNAH6, CFAP70, PROM1, TENM4, CFAP43, DNAI1, TEKT1, DNAAF1, CC2D2A | 3.806 | 5.11E-04 |
| UP_KEYWORDS | Dynein | DNAH12, DNAI1, DNAH3, DNAH6 | 18.479 | 0.046 |
| UP_KEYWORDS | Cytoskeleton | PPP4R3A, DNAH12, DRC3, WASF2, DNAH3, CFAP221, CCDC146, FLNA, SPAG17, DNAH6, DNAI1, TEKT1, DMD, DNAAF1, CC2D2A, CCDC78, SEPT9 | 2.277 | 0.071 |
| UP_SEQ_FEATURE | region of interest:Stem | DNAH12, DNAH3, DNAH6 | 31.846 | 0.263 |
| UP_SEQ_FEATURE | region of interest:Stalk | DNAH12, DNAH3, DNAH6 | 31.846 | 0.263 |
| UP_SEQ_FEATURE | region of interest:AAA 1 | DNAH12, DNAH3, DNAH6 | 31.846 | 0.263 |
| UP_SEQ_FEATURE | region of interest:AAA 3 | DNAH12, DNAH3, DNAH6 | 31.846 | 0.263 |
| UP_SEQ_FEATURE | region of interest:AAA 2 | DNAH12, DNAH3, DNAH6 | 31.846 | 0.263 |
| UP_SEQ_FEATURE | region of interest:AAA 5 | DNAH12, DNAH3, DNAH6 | 31.846 | 0.263 |
| UP_SEQ_FEATURE | region of interest:AAA 4 | DNAH12, DNAH3, DNAH6 | 31.846 | 0.263 |
| INTERPRO | IPR024317:Dynein heavy chain, P-loop containing D4 domain | DNAH12, DNAH3, DNAH6 | 29.934 | 0.234 |
| INTERPRO | IPR013602:Dynein heavy chain, domain-2 | DNAH12, DNAH3, DNAH6 | 28.063 | 0.224 |
| INTERPRO | IPR004273:Dynein heavy chain domain | DNAH12, DNAH3, DNAH6 | 28.063 | 0.224 |
| INTERPRO | IPR026983:Dynein heavy chain | DNAH12, DNAH3, DNAH6 | 28.063 | 0.224 |
| INTERPRO | IPR024743:Dynein heavy chain, coiled coil stalk | DNAH12, DNAH3, DNAH6 | 28.063 | 0.224 |
| KEGG_PATHWAY | hsa05016:Huntington's disease | DNAH12, DNAI1, DNAH3, NDUFC1, DNAH6, SOD2 | 4.153 | 0.825 |
| GOTERM_CC_DIRECT | GO:0005874~microtubule | DNAH12, DNAI1, TEKT1, DNAH3, SPAG17, DNAH6, SEPT9 | 3.362 | 0.249 |
| GOTERM_CC_DIRECT | GO:0005929~cilium | CFAP70, DNAH12, DNAI1, TEKT1, CFAP221 | 4.946 | 0.239 |
| COG_ONTOLOGY | Cytoskeleton | DNAH12, DNAH3, DNAH6 | 9.803 | 0.285 |
| UP_KEYWORDS | Microtubule | DNAH12, DNAI1, TEKT1, DNAH3, SPAG17, DNAH6 | 3.267 | 0.384 |
| UP_KEYWORDS | Motor protein | DNAH12, DNAI1, DNAH3, DNAH6 | 4.517 | 0.470 |
| GOTERM_BP_DIRECT | GO:0007018~microtubule-based movement | DNAH12, DNAH3, DNAH6 | 5.706 | 0.981 |
| GOTERM_MF_DIRECT | GO:0003777~microtubule motor activity | DNAH12, DNAH3, DNAH6 | 5.602 | 0.935 |
| INTERPRO | IPR003593:AAA+ ATPase domain | DNAH12, DNAH3, DNAH6 | 3.302 | 0.969 |
| SMART | SM00382:AAA | DNAH12, DNAH3, DNAH6 | 2.958 | 0.962 |
| INTERPRO | IPR027417:P-loop containing nucleoside triphosphate hydrolase | LRRIQ1, DNAH12, DNAH3, RAB12, SLFN11, PAPSS2, DNAH6, SEPT9 | 1.362 | 0.994 |
| UP_SEQ_FEATURE | nucleotide phosphate-binding region:ATP | DNAH12, DNAH3, SLFN11, PAPSS2, EPHA2, DNAH6 | 0.897 | 1.000 |
| GOTERM_MF_DIRECT | GO:0005524~ATP binding | DNAH12, DNAH3, SLFN11, PAPSS2, EPHA2, NRBP2, DNAH6 | 0.699 | 1.000 |
| UP_KEYWORDS | Nucleotide-binding | DNAH12, DNAH3, RAB12, SLFN11, PAPSS2, EPHA2, DNAH6, SEPT9 | 0.682 | 1.000 |
| UP_KEYWORDS | ATP-binding | DNAH12, DNAH3, SLFN11, PAPSS2, EPHA2, DNAH6 | 0.658 | 1.000 |

**Supplementary Table 6**. Expression of cilia-related genes from NHBE cells exposed to diacetyl or 2,3-pentanedione.

| **Gene** | **Diacetyl** | | **2,3-Pentanedione** | |
| --- | --- | --- | --- | --- |
|  | **Fold Change** | **Padj** | **Fold Change** | **Padj** |
| *TEKT1* | 0.76 | 1.29E-03 | 0.75 | 4.05E-03 |
| *CFAP70* | 0.70 | 1.22E-03 | 0.38 | 1.26E-25 |
| *PROM1* | 0.79 | 7.03E-03 | 0.60 | 1.57E-16 |
| *DNAH12* | 0.65 | 7.07E-05 | 0.43 | 1.88E-22 |
| *DNAI1* | 0.75 | 0.03 | 0.61 | 1.16E-05 |
| *DNAH3* | 0.75 | 9.67E-03 | 0.62 | 4.04E-07 |
| *DNAAF1* | 0.77 | 0.01 | 0.62 | 1.08E-07 |
| *CC2D2A* | 0.77 | 0.03 | 0.71 | 4.78E-04 |
| *CFAP221* | 0.66 | 8.03E-04 | 0.59 | 2.16E-05 |
| *SPAG17* | 0.65 | 1.12E-04 | 0.45 | 7.89E-12 |
| *DNAH6* | 0.73 | 9.70E-03 | 0.48 | 9.77E-18 |

**Supplementary Table 7**. Primer sequences for qPCR validation.

| **Gene** | **Forward Primer (5'-3')** | **Reverse Primer (5'-3')** |
| --- | --- | --- |
| ***ALDH1A3*** | TGAATGGCACGAATCCAAGAG | CACGTCGGGCTTATCTCCT |
| ***CC2D2A*** | AAGATTCAACAGCACCGTCTCT | GGGATGGGTCCCTTAAGAAACTT |
| ***CCDC14*** | ATCTTCAGGAAGGCACACTGG | TCAGAATGGATGGAATAGCCAGAAT |
| ***CCL20*** | AAAAACCATGTGCTGTACCAAGAG | AAAGTTGCTTGCTGCTTCTGATT |
| ***CCNL2*** | GTGCTTCTTGTTAATCCTCTGTCG | GGGACAAAATTCAAGTCAAGACAGT |
| ***CDC20B*** | GATGCTGTGGGAAAGTATCATGC | ACGTTGGCGGAATCTTGACT |
| ***CFAP70*** | AATTCTCTGCTGTCCAAACAAGC | TTATACTAGGTGCCTGCACATCC |
| ***CPXM2*** | GGCCCGTGAAGATGTCAGAG | CACCACGCTCCGTCATAAAAA |
| ***CSF3*** | CTTGTGGCCTATAACTCAGCC | CCCACTCAATCACATAGCCCT |
| ***CXCL6*** | GTTGAGGGAGGTATCCTGTTGTT | GGTATGACACACCCAGTGTTCT |
| ***DHRS9*** | TTCAATGACAGCTTAAGACGGGA | CTTTACTGGATCTGCCAAGTTTGTT |
| ***DNAAF1*** | CCTTGACCTTTCGCACAACAA | CAAATTCAGTACACGCAAATCGG |
| ***DNAH12*** | TCCCAGAAAACATAGGCGTTG | AGAGGTGTTCTTTTACCCAGTGT |
| ***DNAH3*** | CAGATCGCCAAAAGTGACTCC | GTTCCTCATTAGCAGAAGCAGG |
| ***DNAI1*** | GTTGACCGATGCGGAGTTAAA | ACCTGACAATGTTCTGGGGTG |
| ***GAPDH*** | ACAACTTTGGTATCGTGGAAGG | GCCATCACGCCACAGTTTC |
| ***GPX2*** | GGTAGATTTCAATACGTTCCGGG | TGACAGTTCTCCTGATGTCCAAA |
| ***IL33*** | TGAATCAGGTGACGGTGTTGAT | GGTCTGGCAGTGGTTTTTCAC |
| ***KRT13*** | GAGATGGAGTGCCAGAACCAA | TGAGGAAGGGAAACCAATCATCTT |
| ***KRT14*** | TGAGCCGCATTCTGAACGAG | GATGACTGCGATCCAGAGGA |
| ***KRT16*** | GACCGGCGGAGATGTGAAC | CTGCTCGTACTGGTCACGC |
| ***KRT4*** | ACATCAAGAAGCAGTGCCAGA | TGTGGGCATCTTTAAGGGCAT |
| ***MACROD2*** | GAGACTATATTCCCCTGAACAGCAT | GGCATTTGCGGCATTGACTAT |
| ***MIR205HG*** | ATCTCTCAAGTACCCATCTTGGA | GGCCTCATGGTTGTCAGCTC |
| ***PAPSS2*** | CCTGCTAGATGATGGCGTGAT | CCAGGACAAACTTGCTGCAC |
| ***PROM1*** | AGTCGGAAACTGGCAGATAGC | GGTAGTGTTGTACTGGGCCAAT |
| ***PTGS2*** | TCCACCAACTTACAATGCTGACTA | CAGGAAGCTGCTTTTTACCTTTGA |
| ***PTHLH*** | CGACACACGCACTTGAAACTT | TCGGGACCTCCTCTGGTG |
| ***SNHG14*** | TGGATGAGGGTGATGCCTATTAAG | CCCCCGGGTCATGAAAACA |
| ***SORCS2*** | GGCGGTAGTGGTGCTGTT | GGCCGTTTCCTTTTGAACTTGTA |
| ***SPAG17*** | CAGTATGGTGTCGTGGCAAGA | GAGCATTACCACCTACAGGTTTT |
| ***TAX1BP3*** | CTGCGTCAAGGTGAGAACTTAATC | TCAGACACCCGTGTGACATAAAT |
| ***TEKT1*** | CTGCTGACCCGTACCTTGG | TGGTGAGTTGTTGTTGAGCGA |
